# FDG Uptake Pattern on PET/CT Imaging in Non-Infectious Graft of a Patient with Operated Abdominal Aortic Aneurysm

**DOI:** 10.4274/Mirt.98

**Published:** 2012-12-20

**Authors:** Pelin Özcan Kara, Gonca Kara Gedik, Taylan Kara, Ismail Koçak, Erhan Varoğlu, Oktay Sarı

**Affiliations:** 1 Selcuk University Selcuklu Medical Faculty, Department of Nuclear Medicine, Konya, Turkey; 2 Beyhekim State Hospital, Department of Radiology, Selcuklu, Konya, Turkey; 3 Selçuk University Meram Medical Faculty, Department of Nuclear Medicine, Konya, Turkey

**Keywords:** Renal cell carcinoma, positron-emission tomography/computed tomography, 18F-FDG, vascular grafting

## Abstract

Positron emission tomography using fluorodeoxyglucose is a well known diagnostic tool for routine assessment of the patients with carcinoma. Fluorodeoxyglucose uptake, as a marker of glucose metabolism, is increased in malignant conditions as well as infectious and inflammatory processes. In this case report, findings of postoperative changes in the graft on FDG PET/CT were presented in a patient on follow-up for operated renal cell carcinoma and aortic aneurysm graft surgery. The importance of the FDG uptake pattern has been pointed out for differential diagnosis of infectious and non-infectious conditions.

**Conflict of interest:**None declared.

## INTRODUCTION

Flourodeoxyglucose-Positron Emission Tomography/ Computed Tomography (FDG-PET/CT) is an imaging procedure revealing the differences in glucose metabolism. As well as malignant and infectious processes, inflamatory cells also use high amounts of glucose (1). The intensity of foci of inflammation measured on FDG-PET imaging, is correlated with the degree of histopathological measurements (2). FDG-PET is gaining clinical importance in the detection of metabolically active processes in major vascular diseases (3). 

In this case report, we report a sixty-four-year-old man sent to nuclear medicine department for PET/CT imaging for re-staging of renal cell carcinoma. He has gone through left radical nephrectomy and splenectomy and underwent an aorta-bi-iliac grafting two months ago, as well. On FDG-PET/CT imaging diffuse increased FDG uptake at the wall of the abdominal aorta and right and left iliac arteries, from the level of the third lomber vertebra was observed in favour of non-infectious conditions (postoperative changes). The criteria used for considering this uptake pattern as non-infectious condition with a detailed review of literature is clarified in the present report. The importance of FDG uptake pattern has been pointed out for differential diagnosis of infectious and non-infectious conditions.

## CASE REPORT

A sixty-four-year-old man was admitted to hospital because of abdominal pain in the lower left quadrant spreading to the groin two months ago. The transverse diameter of descending thoracic aorta and proximal abdominal aorta were exceeding 3 cm. Aortic aneurysm, fusiform aneurysm in intra-renal abdominal aorta and changes in density due to acute bleeding in thrombus were detected on abdominal CT scan. In addition, in the middle anterior section of left kidney an exophytic, approximately 7x8 cm in size mass with heterogeneous density, consistent with renal cell carcinoma was detected. He had left radical nephrectomy, splenectomy and aorta-bi-iliac grafting 2 months ago for renal cell carcinoma, and was operated for infrarenal abdominal aortic aneurysm. He was sent to nuclear medicine department to perform PET/CT imaging for re-staging. Before the PET/CT examination neither elevation in infection parameters on blood examination nor symptoms pointing an infection was noted. His erythrocyte sedimentation rate, white blood cell count and CRP levels were normal. He had also no clinical signs such as fever, local pain or bacteraemia. 

**Figure 1:**
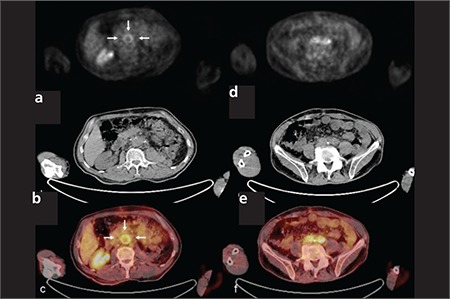
Diffuse increased uptake of FDG on abdominal aorta, rightand left iliac communis arteries on axial PET (a), CT (b) and PET/CTfusion (c) images (SUVmax: 5.4)

**Figure 2:**
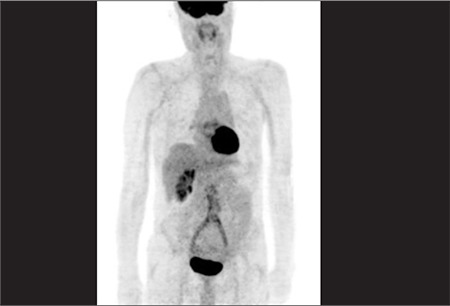
MIP (Maximum intensity projection) image of the samepatient

**Figure 3:**
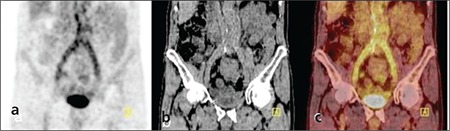
Coronal PET (a), CT (b) and PET/CT fusion (c) images

On PET/CT imaging a 10 mm lymph node with increased FDG uptake in the lower right paratracheal area was observed. In addition, diffuse increased FDG uptake (SUVmax: 5.4) at the wall of the abdominal aorta, right and left iliac arteries from the level of the third lomber vertebra was seen (Figure 1, 2 and 3). FDG uptake in abdominal aortic graft was an additional finding. This uptake pattern was attributed to postoperative changes. Prosthetic vascular graft infection was not suspected in this patient and therefore leukocyte scintigraphy was not performed. On clinical and radiological follow-up the patient showed no further evidence of graft infection. The most important factor in the management of this patient was the differential diagnosis.

## LITERATURE REVIEW AND DISCUSSION

In the treatment of abdominal aorta aneurysm aortic reconstructive surgery with prosthetic graft is commonly used. Graft infection after the operation is a rare but severe complication with high morbidity and mortality. A prompt and accurate diagnosis is important. The clinical presentation is nonspecific and may occur long after surgery. CT scan is the primary imaging modality in graft infections. Perigraft fluid collection, focal bowel wall thickening, pseudoaneurysm formation and ectopic gas are CT findings for graft infection. When CT is negative for infection, leukocyte scintigraphy can be done in second stage. A thoraco-abdominal CT-angiography and a Tc-99m HMPAO labelled leukocyte scintigraphy did not show any graft infection in a case report by Gardet E, et al, whereas FDG-PET scan showed a metabolic uptake around and all along the vascular graft (4). The authors concluded that further comparison between two explorations is needed.

Increased FDG uptake can be seen in leukocytes, activated inflammatory cells such as granulocytes and macrophages. Therefore, PET/CT imaging is a very sensitive imaging modality in the diagnosis of infection. In a few case reports, FDG-PET and PET/CT was found to be a useful diagnostic modality for the evaluation of suspected infected grafts (5,6,7,8). In a case report by Marion MD et al (9), the authors reported the mycobacterium abscessus infection of an infrainguinal vascular bypass prosthetic graft localized by FDG-PET/CT scan. A diameter of ≥ 5.5 cm is the main criterion for abdominal aortic aneurysm repair. Increased FDG uptake in the presence of inflammation was suggested that this may be a better predictor of rupture risk than aneurysm size (10). In a prospective study by Keidar Z et al (11), a total of 39 patients with 69 grafts (range 1-4 grafts/patient) were evaluated using FDG-PET/CT. Infection was suspected on the basis of clinical signs in 40 of 69 grafts. The other 29 grafts were not suspected of being infected. Focal increased FDG uptake in the region of any of the vascular grafts, with intensity higher than that of surrounding tissues, was defined as an infectious process. Studies with no FDG uptake or showing only linear uptake of low to moderate intensity along the graft region were considered negative for the presence of infection. The authors reported a sensitivity of 93%, specificity of 91%, positive predictive value of 88% and negative predictive value of 96% for infection in vascular graft for PET/CT. No abnormal FDG uptake was seen in any of the 29 grafts not clinically suspected of being infected. Two false positive PET/CT results were related to FDG uptake in an infected haematoma adjacent to and surrounding the graft. Mildly increased linear FDG uptake was seen along 10 grafts that had no further evidence of infection.

FDG-PET image may have a role in the diagnosis of infection, but false positive results are possible and caution is necessary if other data are non-confirmatory (12). Postsurgical inflammatory changes, scar tissue and native vessels are the potential false positive findings with increased FDG uptake. In a report by Wasselius et al (13), the authors suggested caution when using FDG-PET/CT to diagnose infection in synthetic vascular grafts because of the apparent risk of false positive results. In a recent study investigating the diagnostic accuracy of FDG-PET, compared with computed tomography scanning suggested that FDG-PET provides useful tool in the work-up for diagnosis of vascular prosthetic graft infection (14). Although, failure to diagnose graft infection is associated with high morbidity, false positive results may lead to unnecessary surgery. In a study on a comparison of FDG PET and CT imaging including 33 patients with arterial prosthetic graft infection (15), when focal FDG uptake was taken as a positive criterion, specificity and positive predictive value of PET were found 95% and 91%, respectively. In this study, false positive FDG uptake has been identified on visual analysis in the 8 of 22 patients with non-infective graft as in our patient. Although the degree of FDG uptake varies in these patients, diffuse and circular FDG uptake was observed in all patients as in our patient. This characteristic uptake pattern is mostly compatible with foreign-body reaction and postoperative changes. While focal FDG uptake is often due to graft infection, in non-infectious cases diffuse pattern of involvement throughout the graft as in this case is observed. The information about the combination of intensity and pattern of FDG uptake, related to the anatomical localization of both pathological tracer uptake, may allow better differentiation between infected and non-infected aortic grafts (16).

FDG uptake in abdominal aortic graft was an additional finding on PET/CT examination in our patient. Prosthetic vascular graft infection was not suspected in this patient and therefore leukocyte scintigraphy was not performed. On clinical and radiological follow-up, the patient showed no further evidence of graft infection. The most important factor in the management of this patient was the differential diagnosis, because aortic prosthetic graft infection in the absence of immediate treatment with antibiotics and surgical intervention is associated with high mortality and morbidity (17). A complete and accurate diagnosis is important in aortic prosthetic graft infection. False positive results may lead to unnecessary surgery. On the other hand, failure to diagnose vascular graft infection is associated with high-risk morbidity. Linear mild to moderate FDG uptake along vascular graft pattern has been attributed to the chronic aseptic inflammation in synthetic graft material (18). This pattern can persist for years after surgery (11,13,15). 

In summary, linear mild to moderate diffuse and circular FDG uptake along vascular graft in recently implanted grafts should not be reported as infection. Combining the anatomic information provided by CT from PET/CT with the presence of increased metabolism on PET improves diagnostic accuracy. Further studies with large number of patients are needed comparing diagnostic performance of PET/CT and leukocyte scintigraphy as a diagnostic cornerstone in vascular graft infections.
